# Age-dependent response to multimodal therapy for stage II-III rectal cancer

**DOI:** 10.18632/oncotarget.27084

**Published:** 2019-07-23

**Authors:** Daniel Delitto, Tyler J. Loftus, Atif Iqbal

**Affiliations:** Department of Surgery, Baylor College of Medicine, Houston, TX, USA

**Keywords:** young, rectal cancer, survival, age

Despite a decreasing overall incidence and mortality for colorectal cancer, it is worsening in younger patients (<50 years of age) [1, 2]. Current clinical guidelines in rectal cancer are developed irrespective of age and represent data extrapolated from trials primarily enrolling older (age >50 years) patients. A differing tumor biology in younger patients may influence response to therapy. We therefore hypothesized that younger patients with rectal cancer may not benefit from the same treatment guidelines as the older population.

To assess this, the National Cancer Data Base (NCDB) was queried for patients who underwent transabdominal resections for stage I–III rectal cancer during a 10-year period. 43,106 patients were included, 13% of whom were less than 50 years old [3]. Younger patients were diagnosed at higher clinical stages (stage I: 33% vs. 39%, stage II: 27% vs. 30%, stage III: 40% vs. 31%, *p* < 0.001). Poorly differentiated and undifferentiated tumors were also more common among younger patients (14% vs. 12%, *p* < 0.001). Younger patients were more likely to receive radiation therapy, including radiation therapy for stage 1 disease (42% vs. 32%, *p* < 0.001) and chemoradiation for stage II and III disease (94% vs. 88%; *p* < 0.001). Younger patients also had longer age-specific survival for all stages of disease. The sentinel finding in this study was that younger patients experienced survival benefit for NCCN guideline-directed therapy for stage I disease, but not stage II or III disease. Following NCCN guideline-directed therapy was associated with decreased survival among patients age less than 45 years (*p* < 0.03). NCCN guideline-directed therapy was associated with a survival benefit at age greater than 54 years.

Further inquiry is critical regarding the biological divergence between rectal cancer in the young versus the elderly. As has been shown in colon cancer, younger patients have a higher incidence of microsatellite instability, though the clinical implications of this finding remain controversial [4, 5]. If rectal cancers or the host response to malignancy in young patients are biologically unique, then tailored treatment recommendations have the potential to improve outcomes, even if the mechanisms remain unknown. For example, excluding chemoradiation for younger patients with early stage rectal cancer may decrease late manifestations of toxicity and future health problems. In addition, prior randomized trials have often excluded younger patients. Evidence that young patients with early stage rectal cancer do not benefit from the current standard of care should serve as a call to arms for the performance of high-quality multicenter randomized trials targeting this knowledge gap. Continued basic and translational scientific rigor may provide evidence of further refinement of NCCN guidelines, toward the ultimate goal of improving the lives of young patients with early stage rectal cancer.
